# Factors controlling soil organic carbon stability along a temperate forest altitudinal gradient

**DOI:** 10.1038/srep18783

**Published:** 2016-01-06

**Authors:** Qiuxiang Tian, Hongbo He, Weixin Cheng, Zhen Bai, Yang Wang, Xudong Zhang

**Affiliations:** 1 State Key Laboratory of Forest and Soil Ecology, Institute of Applied Ecology, Chinese Academy of Sciences, Shenyang 110016, China; 2 Key Laboratory of Aquatic Botany and Watershed Ecology, Wuhan Botanical Garden, Chinese Academy of Sciences, Wuhan 430074, China; 3 Environmental Studies Department, University of California, Santa Cruz, CA, USA; 4 University of Chinese Academy of Sciences, Beijing 100049, China; 5 National Field Observation and Research Station of Shenyang Agro-Ecosystems, Shenyang 110016, China

## Abstract

Changes in soil organic carbon (SOC) stability may alter carbon release from the soil and, consequently, atmospheric CO_2_ concentration. The mean annual temperature (MAT) can change the soil physico-chemical characteristics and alter the quality and quantity of litter input into the soil that regulate SOC stability. However, the relationship between climate and SOC stability remains unclear. A 500-day incubation experiment was carried out on soils from an 11 °C-gradient mountainous system on Changbai Mountain in northeast China. Soil respiration during the incubation fitted well to a three-pool (labile, intermediate and stable) SOC decomposition model. A correlation analysis revealed that the MAT only influenced the labile carbon pool size and not the SOC stability. The intermediate carbon pool contributed dominantly to cumulative carbon release. The size of the intermediate pool was strongly related to the percentage of sand particle. The decomposition rate of the intermediate pool was negatively related to soil nitrogen availability. Because both soil texture and nitrogen availability are temperature independent, the stability of SOC was not associated with the MAT, but was heavily influenced by the intrinsic processes of SOC formation and the nutrient status.

Forests cover 30% of the land area but maintain more than 70% of the global soil carbon. The role of forest soil as a carbon reservoir and source of atmospheric CO_2_ is an important aspect of the global carbon cycle^1^. Because of the low mean annual temperature (MAT) but high soil organic carbon (SOC) content, the carbon release dynamics in temperate forest soils have attracted widespread attention given concerns about climate change. Rising temperatures may not only influence the decomposition of the fresh fraction in soil, but also long-term carbon releases from humified soil organic matter. Therefore, the decomposition of SOC over the long term (referred to here as SOC stability) may offer more significant indications of the potential alteration of carbon release from soil^2^,^3^.

As CO_2_ exchange between soil carbon and atmospheric CO_2_ varies strongly along climate gradients^4^,^5^, many scientists focus on whether there are enhanced response patterns in SOC stability along increasing latitudinal or altitudinal gradients. Numerous studies have implicated temperature as a primary controller of SOC stability by altering the quality and quantity of litter input into soil and soil physico-chemical characteristics^6^,^7^,^8^. SOC stability was found to increase with increasing MAT based on chemical sequential fractionation analysis^9^. However, the components and stability of SOC were not always consistently related to variations on MAT^10^,^11^. Radiocarbon dating and ^13^C enrichment differentiation for soils indicated that SOC stability along latitudinal and altitudinal gradients was negatively related to MAT^5^,^6^,^7^,^12^. Therefore, in addition to temperature affecting SOC stability, other factors must also contribute to SOC stability.

The physical protection of SOC from decomposition, including both spatial inaccessibility of organic matter against decomposer organisms and stabilization by interaction with mineral surfaces, contribute significantly to spatial heterogeneity in SOC stability^13^,^14^,^15^. As a biological process, microbial utilization kinetically determines the extent of substrate decomposition^16^. The metabolic readiness of microorganisms is not merely dependent on the indigenous microbial community structure, which is usually regulated by climate conditions such as temperature^17^, but is also affected by soil nutrient status such as N and P availability due to biological stoichiometry^18^,^19^, which, as a feedback, influences the dynamics of soil organic matter. Furthermore, litter quantity and quality can influence SOC stability by altering the relative abundance of labile and recalcitrant compounds returned to soil^20^. Soil pH and mineralogy are also found to affect soil carbon humification and accumulation^10^,^21^.

Based on the heterogeneity of forest soils^22^, the stability of SOC along climate gradients is likely associated with multiple factors resulting from interactions among physical, chemical and biological processes^10^,^21^,^23^. To understand the relationship between substrate supply and respiratory CO_2_ production during SOC decomposition, one assumes that each soil sample contains a number of carbon pools, each of which has a fixed size and a corresponding decomposition rate^24^,^25^,^26^. In order to determine the factors controlling SOC stability, it is essential to identify the substrate availability, i.e., the sizes of the carbon pools, acting as thermodynamic controls, and their corresponding decomposition rates, acting as kinetic controls^24^,^25^,^26^. By monitoring the accumulated C-CO_2_ released during soil incubation over a certain time period, both the capacity of mineralizable carbon and the decomposition rate can be evaluated according to a first-order kinetic model.

Along an altitudinal gradient (500 m to 2744 m) on Changbai Mountain with the MAT varying from −7.4 °C to 3.6 °C, the initial ground vegetation, soil physico-chemical characteristics and the soil microbial communities are very different^27^. It is not clear whether MAT is the key factor controlling SOC stability and how other factors ultimately determine SOC decomposition dynamics. Therefore, we conducted an incubation experiment on soil samples along the altitudinal gradient and periodically measured soil respiration. A carbon decomposition model based on CO_2_ release dynamics was developed to evaluate the pool sizes and decomposition rates to differentiate the thermodynamic and kinetic control of SOC decomposition. The objective of this study was to 1) investigate how SOC stability changes along the altitudinal gradient; 2) determine the contribution of the different pools to the released carbon; and 3) seek the factors influencing SOC stability along the altitudinal gradient.

## Results

### Soil properties along the altitudinal gradient

Main characteristics of the sites and soils along the altitudinal gradient are given in Tables 1 and 2. The annual amount of litter inputs declined with increased elevation. The annual decomposition rate of litter was lowest at site C and highest at site A. The SOC declined from soil A to soil C with increased elevation but increased from soil C to soil E. The total N concentrations increased significantly from soil B to soil E, except for the highest value in soil A. The C:N ratios in soils A and C were significantly lower than in soils B and E. The proportion of light fraction carbon in the total SOC ranged from 12.2% (soil C) to 25.3% (soil B) but was not correlated with the altitudinal gradient. The proportion of oxidizable carbon in the SOC ranged from 27.0% to 38.4% and declined significantly with increased elevation (*p* < 0.05). The content of soil available N was highest in soil A and lowest in soil B. Soil available P showed no significant differences among the five soils. The content of soil available K in soils C and D was lower than in the other three soils. All five soils were acidic, with pH values ranging from 4.23 to 6.24. The five soils had clay fractions from 12.3% to 21.6%. Soil A had the largest percentage of silt particles, whereas soil E had the largest proportion of sand particles.

The most closely correlated properties involved the soil particle size fractions. Therefore, only the sand fraction was used as an independent factor to represent the soil texture. The soil total N and available N were also significantly correlated with each other, and the soil total N was chosen as a nutrient parameter. Soil C:N and pH were significantly related to the soil texture and total N, thus they remained as dependent factors and were discarded during further analyses.

The relationship between elevation and each of these soil and litter parameters was evaluated using a correlation analysis (data not shown). The mean annual amount of litter was negatively related to elevation (*p* < 0.05). Other parameters, such as soil TN, soil available P, available K, the proportion of sand fraction (sand%), mean annual decomposition rate of litter (k_litter_), litter C:N, and soil microbial biomass were all unrelated to elevation.

### Soil respiration rate

**Figure 1:**
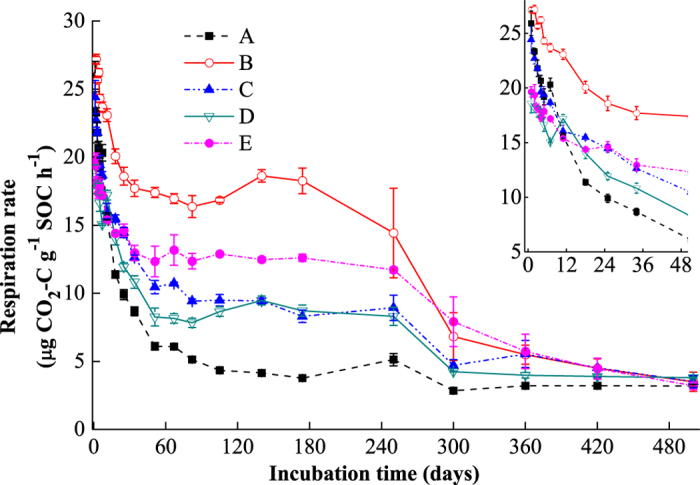
SOC-specific respiration rates (μg CO_2_–C g^−1^ SOC h^−1^) of soil samples at different elevations (A–E) during the 500-day incubation at 15 °C; each point is a mean value (n = 3). The insert represents results during the initial 50-day incubation.

The respiration rates were expressed as μg C-CO_2_ g^−1^ h^−1^ per SOC to remove the effects of different levels of SOC in the five soils and the decreasing content of SOC during the incubation period. The respiration rates of all soil samples declined over the entire incubation period, but the pattern was site-specific and time-dependent (Fig. 1). The respiration rates decreased sharply during the initial 50 days, decreased slowly for the subsequent 250 days, and then remained low and constant for the last 200 days of incubation. At the beginning of incubation, the respiration rates of the five soils were as follows: B > A ≥ C > E ≥ D. Between days 50 and 300, the respiration rates were as follows: B > E > C ≥ D > A. At the end of the incubation, the soil respiration rates exhibited no significant difference across the full elevations.

### Cumulative proportion of respired carbon in SOC

**Figure 2:**
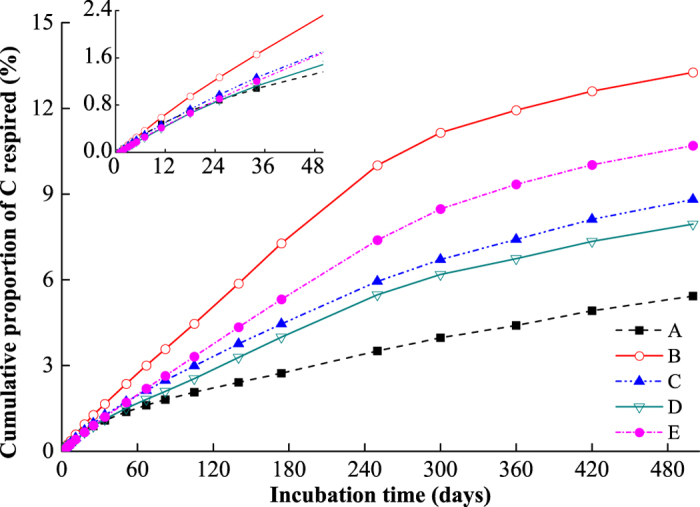
Cumulative proportion of respired carbon (%) of the soil samples at different elevations (A–E) during 500-day incubation at 15 °C; each point is a mean value (n = 3). The insert represents results during the initial 50-day incubation.

After a 500-day incubation, the accumulated respired carbon per dry soil was greatest in soil E (14.94 mg g^−1^ soil), followed by soils B (10.92 mg g^−1^), D (8.53 mg g^−1^), A (6.53 mg g^−1^), and C (5.89 mg g^−1^). To remove the effect of different levels of SOC, the cumulative CO_2_-C was expressed as a proportion of the SOC. For all five soils, the CO_2_-C accumulation increased rapidly until 250 days and slowed during subsequent incubation period (Fig. 2). Over the entire incubation period, approximately 5.1–14.1% of the initial soil carbon was decomposed at the following magnitudes: B > E > C > D > A. However, no correlations were found between the cumulative CO_2_-C and the elevation (r = 0.307, *p* = 0.615) nor any of the other soil or environmental parameters.

### Three carbon-pool model fitting

Soil respiration at 15 °C during incubation fitted well to the three-pool (labile, intermediate and stable) kinetic model through non-linear regressions (Table 3) despite the pulse increase of the respiration rate in soil B^28^. The parameters a0, b0, and c0 were the proportions of the labile, intermediate and stable carbon pools, respectively, in the total SOC, and they represented the size of the individual carbon pools. The proportion of the labile carbon pool was the smallest, accounting for only 0.6–0.86% of the SOC. The proportion of the labile carbon pool was highest in soil A and declined with increased elevation. The percentage of the intermediate carbon pool was approximately 9.0–19.4%. It was lowest in soil A and highest in soils B and E. Most of the SOC existed as a stable pool with a proportion of approximately 79.3–90.1%. The stable carbon pools in soils A, C and D were significantly higher than those in soils B and E. The decomposition rate of the labile carbon pool (k1) was largest in soil D and smallest in soil B. The decomposition rate of the intermediate carbon pool (k2) was smallest in soil A, followed by soil E. The magnitude of k3 for all soil samples was smaller than 2 × 10^−5^ day^−1^.

The contributions of each carbon pool to the cumulative released C-CO_2_ after 50-day, 250-day, and 500-day incubation are shown in Table 4. During the 50-day incubation, the labile, intermediate, and stable carbon pool contributed 3.6–56.4%, 41.6–93.3% and 0.9–3.2% to the released carbon, respectively. With increased incubation time, the contribution of the labile carbon pool decreased, and the contribution of the intermediate and stable carbon pools increased. After the 500-day incubation, the intermediate carbon pool contributed more than 90% to the released carbon, except from soil A, in which 79.1% of the carbon release originated from the intermediate carbon pool and 15.9% of the carbon release originated from the labile carbon pool. The contribution of the stable pool was lower than 5.4% for all of the soils.

Correlation analyses were used for each carbon pool size and decomposition rate with physically, chemically and biologically related variables, including the MAT, soil TN, soil available P and available K, sand%, annual litter amount, k_litter_, litter C:N, and soil microbial biomass (Table 5). The labile carbon pool size was significantly negatively related to the MAT (r = 0.880, *p* = 0.049), whereas the decomposition rate of the labile carbon pool was negatively related to the litter C:N (r = −0.909, *p* = 0.003). Although no significant relationship was found for the intermediate carbon pool size with these variables, the sand% was most highly correlated with the intermediate carbon pool size (r = 0.778, *p* = 0.121). The decomposition rate of the intermediate carbon pool had a negative relationship with the soil TN (r = −0.878, *p* = 0.05). The stable carbon pool size exhibited no significant relationship with these variables. A correlation analysis was not developed for k3, because k3 was calculated according to the MAT.

## Discussion

### SOC stability along the altitudinal gradient

Although elevation was the key driver of variation in climate properties, the SOC decomposition during the 500-day incubation had no significant relationship with elevation on our study mountain. In previous research, the climate conditions negatively or positively influencing the SOC stability were primarily determined according to three processes. First, temperatures influenced the process of SOC humification and stabilization^29^,^30^, resulting in differences in chemical stability. Second, annual litter amounts and litter decomposition rates increase positively with MAT. With higher litter amounts and annual litter decomposition rates, most of the labile material is consumed quickly, leaving behind microbial derived stable compounds and more recalcitrant detritus to soil, which ultimately lead to higher biochemical stability of SOC at lower elevations^20^. Third, the soil texture may vary along the altitudinal gradient and lead to the differentiated physical protection of SOC^5^.

In our current research, the three processes regarding to elevation did not occur uniformly. The annual litter amount was negatively related to elevation (r = −0.960, *p* = 0.010), and the biochemical stability of SOC at a lower elevation was supposed to be higher due to the more recalcitrance detrital inputs^20^. However, the soil oxidizable carbon proportion (as an indicator of SOC chemical stability) was significantly negatively related to elevation (r = −0.926, *p* = 0.024), which indicated that the chemical stability of soil carbon was enhanced at higher elevations. Conversely, the proportion of sand particles was positively related to elevation (r = 0.775, *p* = 0.124), indicating that physical stability was weakened at higher elevations. Due to the mixed effects of these processes, the SOC stability along the altitudinal gradient was compromised in our research area.

### Pool-contribution during SOC decomposition and the determinant role of the intermediate carbon pool

During the 500-day incubation, the dynamic of soil respiration rates was divided into three processes (Fig. 1): the respiration dropped dramatically at first, then declined gradually, and at last remained low and stable for the latter period. This respiration dynamics verified the presence of several different pools of organic carbon with different decomposition rates. The differences in the three processes for the five soils were compared and interpreted through a first-order three-pool kinetic model. The initially sharp drop in respiration rates for all of the soils was probably driven by the consumption of the labile SOC pool^2^. Because of the lower decomposition rate of the intermediate carbon pool, the consumption of the intermediate carbon pool was much slower than the labile carbon pool, causing the respiration rates to decrease much more slowly. Following the consumption of the intermediate SOC pool, the respiration rates remained low and stable for the latter period.

The size of the labile soil carbon pool and its decomposition constant was significantly related to the MAT and litter C:N, respectively (Table 5). The labile carbon in forest soils mainly comes from plant residues and root exudates. Compared with other carbon pools, the labile pool was more vulnerable to environmental change and litter properties. With increasing MAT, increasing plant residues may increase carbon replenishment of the labile carbon pool. In this way, the labile carbon pool size would decrease with increased elevation. Simultaneously, the labile carbon that originated from higher quality litter (lower litter C:N) would subsequently demonstrate a higher decomposition rate. In fact, the climate conditions along the altitudinal gradient drove the dynamics of litter quality and quantity in this study; therefore, altitude influenced more essentially the labile carbon pool, rather than the whole SOC pool in this mountainous system. Despite having the largest decomposition rate, the labile carbon pool was smallest in size and thus contributed a negligible proportion to the total C-CO_2_ release (Table 4).

The stable carbon pool constituted the largest proportion of total SOC (approximately 79.3–90.1%). The much lower respiration rate during the later incubation period indicated its dominant contribution to the accumulation of SOC with a resident time of possibly hundreds to thousands of years^31^. According to the statistical analysis, when the magnitude of the decomposition rate (such as k3) was on or less than an order of 10^−5^, it exhibited a negligible effect on the first-order three-pool kinetic model in the 500-day incubation^32^.

The intermediate carbon pool made up 9.0–19.4% of the total SOC, but its contribution to carbon release after 500-day incubation ranged from 79.1–94.9%, suggesting a dominant controlling role on SOC stability. According to the correlation analysis (Table 5), the intermediate carbon pool was not dependent on the MAT. The size of the intermediate soil carbon pool was mostly related to the percentage of the sand fraction. After rapid consumption of the labile carbon in the soil, substrate accessibility became the limiting factor of SOC biodegradability^33^,^34^. As aggregation in the incubated soils was partly disturbed when being ground, the interaction of SOC and minerals was the critical controlling factor for the intermediate carbon pool size that could be potentially decomposed. Compared to the small particle fractions, the sand fraction has lower SOC absorption capacity, thereby causing SOC to become more decomposable^35^,^36^. Our results indicate that the soil texture could influence the allocation of carbon across the intermediate and stable pools in the Changbai mountainous system.

As a kinetic parameter, the SOC decomposition rate was, in general, linked to the ability of microorganisms to utilize substrates^16^. However, the microbial biomass exhibited no influence on the decomposition kinetics of the carbon pools on Changbai Mountain. The soil N content was negatively related to the decomposition rate of the intermediate pool (Table 5), possibly due to progressive N limitation (from the value of the soil C:N ratio in Table 2)^37^. According to the “microbial N mining” hypothesis, nutrient deficiency in soil could enhance microbial activity to acquire N from more stable organic matter^38^. On the other hand, greater N availability could inhibit the activity of oxidative enzymes^39^,^40^,^41^ and weaken the decomposition of organic polymers or lignin and its derivatives, which are usually considered to be the important components of the intermediate or stable carbon pool.

### Soil intrinsic processes controlling SOC stability

In addition to the conspicuous change in temperature along altitudinal gradients in mountainous ecosystems, the physical, chemical and biological properties of soils also change significantly^42^,^43^. Therefore, the SOC stability would be influenced by the confounding factors of climate and site characteristics, but would not be consistently related to temperature variations along the studied altitudinal gradient^10^. Environmentally related factors, such as climate conditions and litter properties, only influenced the labile soil carbon pool, resulting in variation in soil respiration during the early incubation period.

Due to the controlling role of soil texture and N availability on the size and decomposition rate of the intermediate carbon pool, the SOC stability on Changbai Mountain did not exhibit a significant altitudinal effect. The soil texture was shown to significantly moderate soil carbon stability in temperate forest soils in many studies^5^,^33^,^44^. We further found that the interaction between the SOC and minerals determined the capacity of the intermediate pool in a thermodynamic pattern, implying that physical protection might be a universal controlling factor for SOC decomposition readiness. Comparatively, the effect of nutrient status on the stability of SOC might be site dependent in different ecosystems, considering its controlling effect on decomposition kinetics. For instance, N availability has been widely recognized as influencing the decomposition of SOC^23^,^37^,^45^, whereas K and P availability could also affect forest SOC decomposition^23^,^46^. Furthermore, all five soils were incubated at 15 °C despite the variations in MAT. Soils from the highest elevations, such as Alpine tundra (with a mean temperature in the growing season 9.9 °C), experienced a significant temperature rise during incubation. However, the carbon decomposition of Alpine tundra soil remained lower than soil from *Picea* and *Abies* forest, with a mean growing season temperature of 13.4 °C. This highlights the dominant effect of soil properties over temperature on SOC stability.

## Conclusions

The stability of SOC along the altitudinal gradient on Changbai Mountain was evaluated by monitoring the dynamics of soil respiration during a 500-day incubation and fitting with a first-order three-pool kinetic model. Our results indicated that only the labile carbon pool size, not SOC stability, was affected by the MAT. The stability of SOC along the altitudinal gradient was mainly dependent on the intrinsic processes of SOC formation and nutrient status. Because soil N availability can kinetically affect SOC stability, the influence of N deposition on SOC stability should be given more attention. Given that the CO_2_-C release from soil over hundreds of days was derived mainly from the intermediate carbon pool, this pool may become a potential carbon source.

## Materials and Methods

### Site description and soil sampling

The soil samples were collected in the Changbai Mountain National Nature Reserve (41° 58′–42° 06′ N, 127° 54′–128° 08′ E). With increased elevation from 500 m to 2744 m, the MAT decreased from 3.6 °C to −7.4 °C, and the mean annual precipitation increased from 720 mm to 1400 mm. The distinct changes in climate formed a clear vegetation gradient along the altitudinal gradient: *Pinus koraiensis* and broadleaf mixed forest (500–1100 m), *Picea* and *Abies* forest (1100–1600 m), *Larix* and *Abies* forest (1600–1800 m), *Betula ermanii* forest (1800–2000 m), and Alpine tundra (2000–2744 m). In this study, five soil samples were collected from the mineral soil layers (A horizon) along an altitudinal gradient on the northern slope of Changbai Mountain in the summer of 2010. Detailed site information is provided by Tian *et al*.^28^ and is summarized in Table 1. Here, A, B, C, D, and E represent the five soils from low to high elevations. The data of annual amount and decomposition rate of forest litter are from Liu *et al*.^47^ and Liu *et al*.^48^. The litter data of Alpine tundra are from Wei *et al*.^49^. Soil samples were collected from three randomly chosen locations within each site. All of the soil samples were brought to the laboratory and passed through a 2 mm sieve immediately after the visible roots and stones were removed. The soil microbial biomass was measured using these fresh soils as promptly as possible. Field replicates were homogenized and stored air-dried until incubation began.

### Laboratory incubation

To evaluate SOC stability in this mountainous system, laboratory incubation was conducted for 500 days with the incubation temperature set at 15 °C, which is approximately the mean temperature in the growing season at the five sites. For each soil, 6 replicate samples (25 g dry soil per sample) were weighed and placed in plastic specimen bottles (150 ml, 3 cm diameter). All of the soil samples were pre-incubated for 15 days at 15 °C to allow the soil to equilibrate after sieving and handling. The moisture of the soil samples was initially adjusted to and maintained at 65% of the water holding capacity (WHC) through the addition of deionized H_2_O at regular intervals (1–2 weeks). The WHC was determined by saturating a sample of soil in filter paper placed in a glass funnel. Then, the water was drained for 2 h before the gravimetric soil moisture content (for 100% WHC) was determined by drying for 24 h at 105 °C.

### Soil respiration measurements

The respiration rates of the soil samples were assayed daily for the first week, every four days for the next three weeks, weekly for the second month, and monthly for the subsequent incubation time at 15 °C. At each time period, three of the six replicates were randomly used to measure the soil respiration. The released CO_2_ was measured by connecting a Li-COR IRGA 6262 (Li-COR Biosciences, Lincoln, NB, USA) and a mass flow meter to the outflow tube of each soil sample according to the method described by Tian *et al*.^28^, and then the flow rate and CO_2_ concentration were recorded.

### Soil property determination

The content of SOC and total nitrogen (TN) was determined using an elemental analyzer (Model CN, Elementar Analysensysteme GmbH, Hanau, Germany). The content of SOC for soils during incubation was calculated from the SOC content at the beginning of incubation, subtracting the cumulative amount of C-CO_2_ released. The soil pH was measured with a calomel electrode on a paste of 1:2.5 (w:v) of air-dried soil and deionized water. The soil texture was determined with a Bouyoucos hydrometer and the relative proportions of sand, silt and clay were ascertained gravimetrically from the settling time of the soil suspension. The soil available N was determined using an alkaline hydrolysis method described by Wang *et al*.^50^. The soil available P was extracted with 0.03 mol L^–1^ NH_4_F and was detected based on its absorbance on a spectrophotometer at 700 nm. The soil available potassium (K) was extracted with 1.0 mol L^–1^ NH_4_OAc and was measured using emission flame spectrometry.

To estimate the chemical stability of SOC, density fractionation was performed using a sodium polytungstate solution^14^ with a density of 1.6 g cm^−3^ as an agent to separate the light and heavy fractions. Each fraction was analyzed for carbon and N content as described above. Furthermore, the chemical oxidized fractionation was determined in 330 mmol L^–1^ KMnO_4_^51^. The soil microbial biomass was estimated through substrate induced respiration according to Anderson and Domsch^52^.

### Calculations and statistics

The respiration rates of the soil samples were calculated based on the formula developed by^53^:
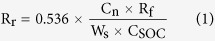
where R_r_ is the respiration rate normalized to SOC in units of μg C-CO_2_ g^−1^ SOC h^−1^; C_n_ is the CO_2_ concentration in mmol CO_2_ mol^−1^; R_f_ is the flow rate in mL h^−1^; W_s_ is the gram dry weight of the soil sample; and C_SOC_ is the content of SOC in mg g^−1^.

The cumulative amount of C-CO_2_ released was calculated by integrating the soil respiration rate with time. On the basis of the cumulative amount of CO_2_-release during the incubation and the nonlinear least-squares regression by the Marquardt algorithm, a first-order three-pool kinetic model was fitted to partition the SOC into labile, intermediate and stable carbon pools and was presented as^24^,^25^,^26^:
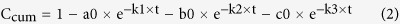
where C_cum_ is the cumulative amount of C-CO_2_ released at time t, which is expressed as a portion (%) of SOC; a0, b0, and c0 are the respective portions of the labile, intermediate and stable SOC pools in SOC (a0 + b0 + c0 = 1); and k1, k2, and k3 (day^−1^) are the respective decomposition rates for the labile, intermediate and stable SOC pools. Radiocarbon dating of non-hydrolysable carbon indicated that the residue time of this recalcitrant fraction was about thousand years^31^. Statistical analysis also indicated that when k3 was very small (<5 × 10^−5^), there was little effect on the first-order three-pool kinetic model^31^. To obtain a valid convergence fitting value, we assumed that the resident time of stable carbon pool in the field was on the order of one thousand years, and that k3 was calculated according the temperature difference between the MAT and our incubation temperature^54^.

The proportion of the labile, intermediate, and stable carbon pools contributing to the 50-day, 250day, and 500-day cumulative released carbon was calculated as follows:
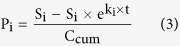
where P_i_ is the contribution of the i pool to the released C-CO_2_ (i is labile, intermediate or stabile SOC pool); S_i_ is the pool size of the i pool; k_i_ is the decomposition rate of the i pool; t is the incubation time, and C_cum_ is the cumulative amount of C-CO_2_ released after 50-day, 250-day, and 500-day incubation.

Correlation analysis was used to search the potential factors affecting the carbon pool sizes and decomposition rates. The factors included the environmental temperature represented as the MAT; the relevant soil characteristics (e.g., soil TN, C:N, pH, particle size fraction, and soil available N, P, and K); litter properties (e.g., the mean annual litter amount, annual decomposition rate (k_litter_), and C:N of litter); and the soil microbial biomass. Before further analysis, some co-related variables were removed using a correlation analysis.

The significant differences among the physical and chemical soil properties were analyzed using a one-way analysis of variance (ANOVA). Significant differences in the soil respiration rates and the amounts of accumulated respired carbon during incubation were determined using repeated measurements analysis of variance. The differences were considered statistically significant at *p* < 0.05 with Tukey’s HSD as post hoc. ANOVA and correlation analyses were performed using SPSS 16.0 (Chicago, IL, USA). Nonlinear least-squares regression was performed using R version 3.1.1.

## Additional Information

**How to cite this article**: Tian, Q. *et al*. Factors controlling soil organic carbon stability along a temperate forest altitudinal gradient. *Sci. Rep.* **6**, 18783; doi: 10.1038/srep18783 (2016).

## Figures and Tables

**Table 1:** Site descriptions along an altitudinal gradient on the northern slope of Changbai Mountain.

**Description**	**A**	**B**	**C**	**D**	**E**
Elevation (m)	791	1247	1707	1975	2294
MAT (°C)	3.6	0.5	−2.0	−3.2	−7.4
Mean temperature in the growing season (°C)	15.75	13.38	12.29	11.52	9.95
MAP (mm)	700	800	1000	1074	1120
Annual amount of litter (g m^2^)	490	450	330	280	115
Annual decomposition rate of litter	0.55	0.49	0.26	0.45	0.35

A: *Pinus koraiensis* and broadleaf mixed forest (791 m); B: *Picea* and *Abies* forest (1247 m); C: *Larix* and *Abies* forest (1707 m); D: *Betula ermanii* forest (1975 m); and E: Alpine tundra (2294 m).

**Table 2:** Characteristics of the sampled soils along the altitudinal gradient on the northern slope of Changbai Mountain.

**Characteristic**	**A**	**B**	**C**	**D**	**E**
SOC (mg g^−1^)	127	77.2	65.5	110	135
Soil TN (mg g^−1^)	8.94	3.89	4.98	5.53	6.73
C:N	14.2	19.9	13.2	20.0	20.0
Light fraction carbon/SOC (%)	20.0	25.3	12.2	12.3	23.3
Oxidizable carbon/SOC (%)	38.4	32.4	30.3	31.5	27.0
Microbial biomass (mg C g^−1^ soil C)	24.5	17.0	32.2	10.8	22.2
pH (1:2.5 soil:water)	6.24	4.64	4.61	4.23	4.54
Soil available N (μg g^−1^)	540	273	421	407	490
Soil available P (μg g^−1^)	15.1	14.5	9.33	8.51	10.7
Soil available K (μg g^−1^)	282	294	200	241	294
Sand% (2–0.05 mm)	15.1	39.8	27.1	37.4	47.2
Silt% (0.05–0.002 mm)	63.3	45.1	53.3	47.5	40.6
Clay% (<0.002 mm)	21.6	15.1	19.5	15.1	12.3

The soil microbial biomass was determined using freshly collected soils. Other characteristics were determined using air-dried soils before incubation. A, B, C, D, and E represent the five soils from low to high elevations.

**Table 3:** The values of the parameters obtained by fitting a three-pool carbon decomposition models for the soil samples at different elevations.

**Sample**	**Fitting parameters**					
	**a0 (%)**	**b0 (%)**	**c0 (%)**	**k1 (day**^−**1**^** × 10**^−**2**^)	**k2 (day**^−**1**^** × 10**^−**3**^)	**k3 (day**^−**1**^** × 10**^−**5**^)
A	0.86	9.0	90.1	4.38	1.29	0.60
B	0.30	19.4	79.3	1.32	2.42	0.75
C	0.24	11.2	88.6	7.32	2.66	0.89
D	0.11	10.2	89.7	12.6	2.69	0.97
E	0.06	18.5	81.4	9.35	1.77	1.29

a0, b0, and c0 are the proportions of labile, intermediate and stable carbon pools and k1, k2, and k3 are the decomposition rates for the labile, intermediate and stable pools of organic carbon, respectively. A, B, C, D, and E represent the five soils from low to high elevations. The residual standard errors of the fitted models for soils A, B, C, D, and E were 0.0003, 0.0038, 0.0005, 0.0010, and 0.0026 with 16 degrees of freedom, respectively. For the value of each fitted parameter, a T-test for the null hypothesis indicated that *p* < 0.05 for all values, except k1 in soils B and E (*p* > 0.05).

**Table 4:** The proportion of the labile, intermediate, and stable carbon pools contributing to the cumulative carbon released after 50-day, 250-day, and 500-day incubation for the soil samples at different elevations.

**Sample**	**50-day (%)**			**250-day (%)**			**500-day (%)**		
	**Labile pool**	**Intermediate pool**	**Stable pool**	**Labile pool**	**Intermediate pool**	**Stable pool**	**Labile pool**	**Intermediate pool**	**Stable pool**
A	56.4	41.6	2.0	24.7	71.4	3.9	15.9	79.1	5.0
B	4.3	65.3	0.9	2.8	86.0	1.4	2.0	89.5	1.9
C	14.2	83.5	2.4	4.1	92.5	3.4	2.7	92.8	4.4
D	7.5	89.5	3.0	2.0	93.9	4.1	1.3	93.3	5.4
E	3.6	93.3	3.2	0.9	95.3	3.8	0.5	94.9	4.6

A, B, C, D, and E represent the five soils from low to high elevations.

**Table 5:** Correlations between the carbon pool and environmental factors that potentially affect the SOC decomposition process.

	**a0**	**b0**	**c0**	**k1**	**k2**	**k3**
MAT	**0.880**	−0.417	0.346	−0.672	−0.273	**—**
	**(0.049)**	(0.485)	(0.568)	(0.214)	(0.657)	**—**
Soil total N	0.656	−0.478	0.464	0.090	**−0.878**	**—**
	(0.229)	(0.415)	(0.431)	(0.885)	**(0.050)**	**—**
Soil available P	0.757	0.204	−0.275	−0.869	−0.643	**—**
	(0.138)	(0.743)	(0.655)	(0.056)	(0.242)	**—**
Soil available K	0.213	0.587	−0.617	−0.414	−0.632	**—**
	(0.730)	(0.298)	(0.267)	(0.488)	(0.252)	**—**
Sand	−0.873	0.778	−0.733	0.297	0.366	**—**
	(0.053)	(0.121)	(0.159)	(0.628)	(0.545)	**—**
K_litter_	0.633	−0.110	0.055	−0.391	−0.468	**—**
	(0.252)	(0860)	(0.930)	(0.516)	(0.427)	**—**
Litter C:N	0.533	0.434	−0.502	**−0.909**	−0.410	**—**
	(0.355)	(0.465)	(0.389)	**(0.033)**	(0.493)	**—**
Microbial biomass	0.241	−0.114	0.112	−0.236	−0.246	**—**
	(0.696)	(0.855)	(0.858)	(0.703)	(0.690)	**—**

Pearson’s correlation coefficients (range from −1 to +1) are followed in parentheses by *p* values (pointing to the significance of the estimated correlations, n = 5). The highest correlations (*p* < 0.05) are presented in bold. a0, b0, and c0 are the portions of the labile, intermediate and stable carbon pool sizes and k1, k2, and k3 are the decomposition rates for labile, intermediate and stable pools of organic carbon, respectively. The MAT is the mean annual temperature; Sand is the proportion of sand particles in the soil; k_litter_ is the annual decomposition rate of litter. A correlation analysis was not developed for k3, because k3 was calculated based on the MAT.
